# Supplementary figures and images for: Transcriptomic Analysis of Spleen Revealed Mechanism of Dexamethasone-Induced Immune Suppression in Chicks
Source: Genes (Basel). 2020 May 6;11(5):513. doi: 10.3390/genes11050513 (PMC7288455; doi:10.3390/genes11050513)

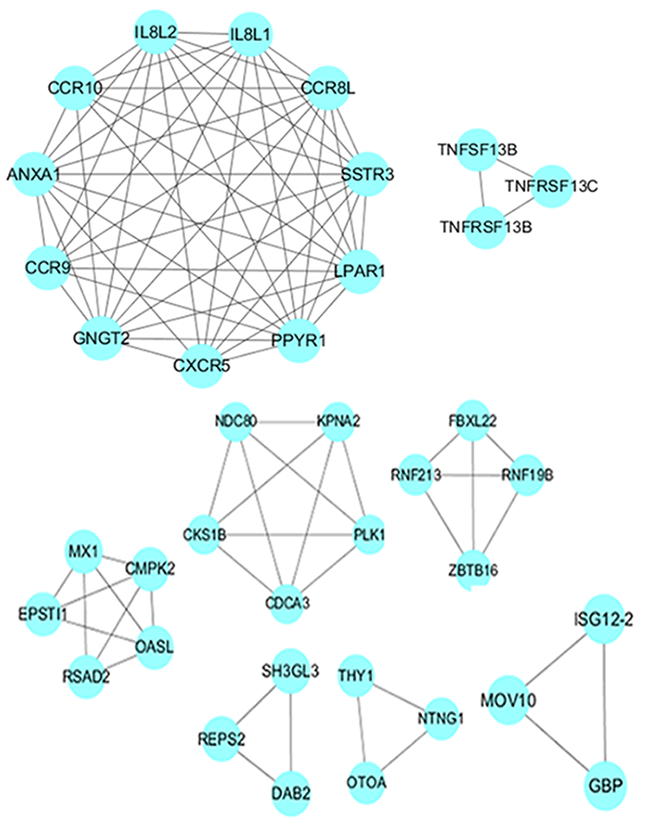

Supplement: Supplementary file 1 [file genes-11-00513-s001.zip › Supplementary Files/Additional File 10 Figure. S3.tif]

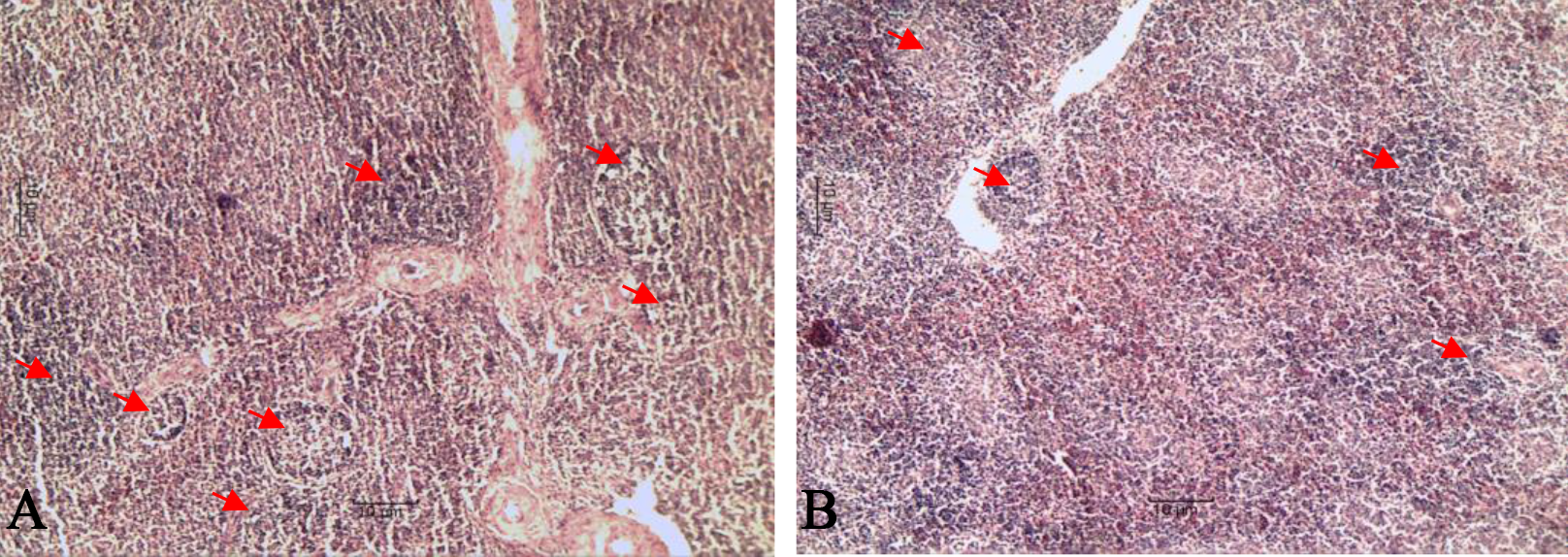

Supplement: Supplementary file 1 [file genes-11-00513-s001.zip › Supplementary Files/Additional File 11 Figure. S4.tif]

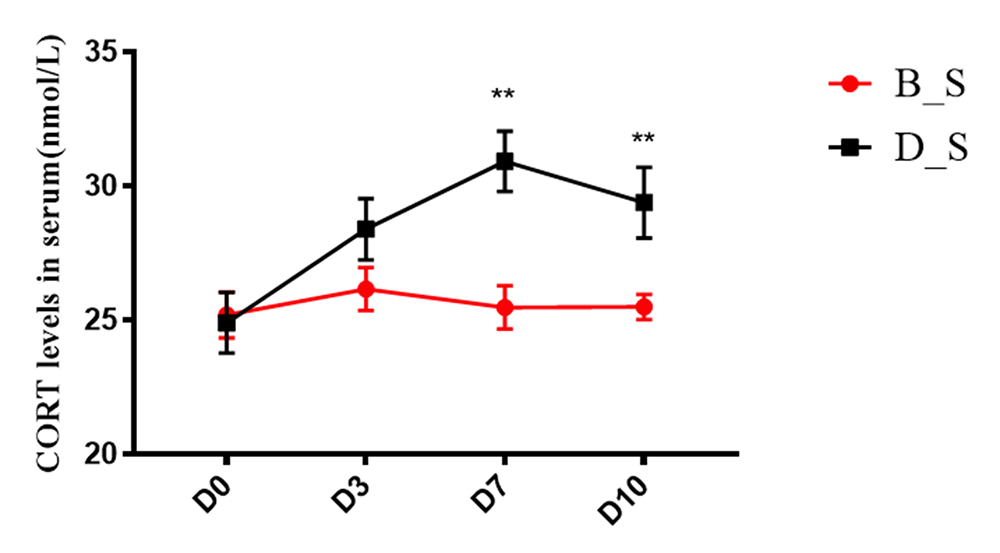

Supplement: Supplementary file 1 [file genes-11-00513-s001.zip › Supplementary Files/Additional File 2 Figure. S1.tif]

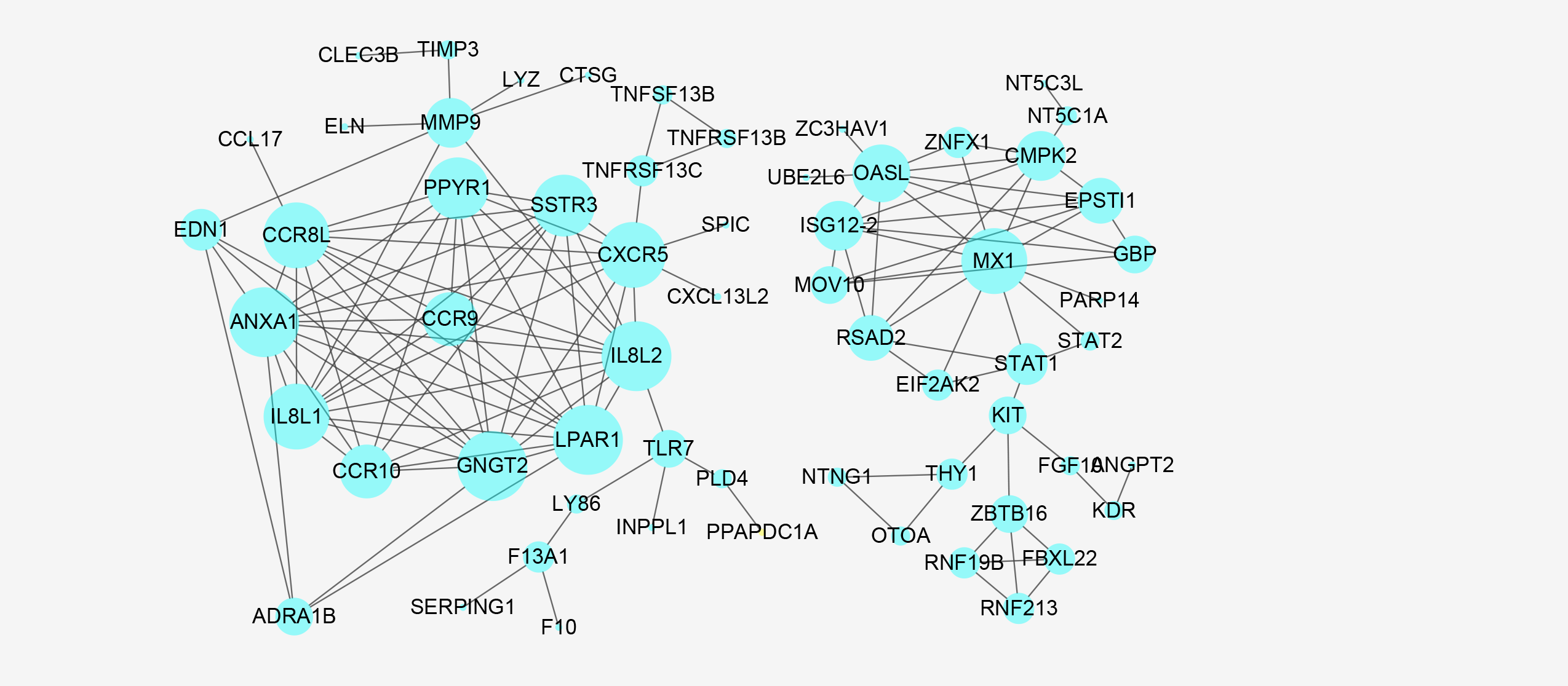

Supplement: Supplementary file 1 [file genes-11-00513-s001.zip › Supplementary Files/Additional File 9 Figure. S2.tif]
